# Editorial: The Interplay Between Social Determinants of Health and Cancer Related Health Disparities

**DOI:** 10.3389/fpubh.2022.887847

**Published:** 2022-05-05

**Authors:** Tung-Sung Tseng, Chien-Ching Li, Alicia K. Matthews

**Affiliations:** ^1^Program of Behavioral and Community Health Sciences, Louisiana State University Health Sciences Center at New Orleans, New Orleans, LA, United States; ^2^Department of Health Systems Management, Rush University, Chicago, IL, United States; ^3^Department of Population Health Nursing Science, University of Illinois Chicago, Chicago, IL, United States

**Keywords:** cancer, health disparities, social determinants of health, cancer inequalities, cancer prevention and control

Cancer is the second leading cause of death among adults in the United States. Since the initiation of the “War on Cancer” by Richard Nixon in 1971, advancements in cancer screening modalities and treatment approaches have resulted in marked improvements in cancer-related outcomes. However, not all groups have benefitted equally from advancements in the diagnosis and treatment of cancer. Indeed, we see persistent inequalities across the cancer care continuum, including prevention, screening, diagnosis, treatment, survivorship, and end-of-life care. Population groups at elevated risk for cancer inequalities include socioeconomically disadvantaged populations, underserved rural populations, racial/ethnic, and sexual and gender minorities. Against this backdrop, President Barak Obama signed into legislation the Cancer Moonshot initiative, which aimed to reduce cancer inequalities by making more therapies available to more patients and improving cancer prevention and early detection efforts. In recent years, emphasis has been placed on understanding and addressing the role of social determinants of health (SDOH) on cancer-related health disparities. A better understanding of SDOH can provide researchers and health professionals with effective strategies for reducing cancer-related health disparities and promoting cancer prevention and control.

The World Health Organization's Commission on the Social Determinants of Health has defined SDOH as factors in which people are born, grow, work, live, and age, and the broader set of forces and systems shaping the conditions of daily life that influence health outcomes (1). An extensive body of research has demonstrated that SDOH have an important influence on health inequities (2). Research, guided by multilevel approaches (e.g., the social-ecological model), has started to be conducted which examines the relationships between SDOH (e.g., income, education, health knowledge and behavior, access to health care, housing, poverty, neighborhood safety, economic stability, political conditions) and cancer-related health disparities (3–5). In this Research Topic of Frontiers in Public Health, “*The Interplay Between Social Determinants of Health and Cancer Related* Health Disparities,” we solicited articles reviewing and addressing the role of social determinants of health in cancer-related health disparities to give readers an overview of updated health disparities information for their potential use in cancer research and practice. The goal of the Research Topic was to address cancer-related disparities with multilevel approaches, measurable outcomes, and effective solutions focused on innovative and effective strategies to understand the impact of SDOH or improve cancer-related outcomes in diverse populations.

This Research Topic collected 12 studies examining the relationship between SDOH and cancer prevention, treatment, survivorship, and disparities. The majority of research focused on the analyses of individual-level SDOH (e.g., race, gender, marital status, income, insurance, health risk behaviors), followed by community-level (e.g., proximity to care, neighborhood poverty), and interpersonal-level (e.g., social support) SDOH. Several studies incorporated multi-dimensional SDOH at the individual, interpersonal, and community levels in the analysis to better understand cancer-related outcomes and disparities. The findings from the collected articles are summarized as follows.

First, two studies examined the relationship of individual-level SDOH on health outcomes and survivorship among cancer survivors by analyzing national representative datasets. Su et al. analyzed the Behavioral Risk Factor Surveillance System survey data. Results showed that lower family income was associated with cancer survivors' poor mental and physical health. Ishino et al. analyzed survivorship outcomes among colorectal cancer patients using SEER data. Older age at diagnosis, female, widowed, and non-Hispanic White with localized staging were associated with the lowest survivorship class.

Second, two studies examined the influence of individual and community-level SDOH on receipt of cancer-related surgery and treatment. Hu et al. analyzing the Surveillance, Epidemiology, and End Results (SEER) data, found higher rates of refusing recommended surgery among Asian/Pacific Islander and African American patients than white patients. Furthermore, racial disparities in the receipt of timely lung cancer surgery were identified among Louisiana patients by Neroda et al.. Black patients were more likely to have delayed surgery than white patients. Other SDOH (insurance, social support, community poverty level) were also associated with receiving timely surgery.

Third, three studies discussed multilevel SDOH associated with cancer screening utilization. Li et al. found that access to care factors (insurance type, proximity to care) were associated with the uptake of low dose computed tomography (LDCT) among screening eligible patients in an academic medical center. Performing multilevel analysis using Swiss nationwide data, Jolidon et al. found that higher income and married women were significantly associated with higher mammography uptake. Beyond individual factors, Tsui et al. found that the health-related social needs assessment (HRSN) could be conducted at the health care organizational level to improve cancer screening utilization in the Chinese American population.

Fourth, other studies focused on identifying the mechanisms by which SDOH contributes to cancer risks, cancer disparities, and the development of cancer prevention strategies. Zhou et al. analyzed the patterns and trends of disease burden and risk factors attributable to ovarian cancer across age, socio-demographic index, regions, and countries in terms of cancer risks. Tseng T-S. et al. indicated that increased cigarette smoking and other risky behaviors (increased sugary drink consumption, spending more time on screens, decreased physical activity time, and sleeping less) were found among African Americans eligible for LDCT screening during the COVID pandemic. Two articles discussed the barriers and facilitators for accessing smoking cession services and treatments in cancer prevention strategies. Tseng T.S. et al. found that geographical distance was a significant predictor of attendance of smoking cessation counseling classes. Shorter traveling distances were associated with more class attendance. Matthews et al. developed a model for developing and implementing smoking cessation interventions via patient health portals for low-income patients to facilitate patient linkage to receive free telephone-based smoking cessation counseling. Lastly, Gehlert et al. use the Critical Race Theory to address breast cancer disparities at the population level with an emphasis on social factors (e.g., race and discriminatory public policies, attitudes toward healthcare by significant others, training for health providers and professionals, availability of preventive services in communities).

Social determinants of health at various levels (individual, interpersonal, community level) have shown impacts on cancer-related health disparities in existing literature. The Centers for Disease Control and Prevention (CDC) have emphasized the SDOH and promoted SDOH data, research, tools for action, programs, and policy to improve health disparities (https://www.cdc.gov/socialdeterminants/index.htm) (6). President Joe Biden's new national goal for the reignited Cancer Moonshot—is to cut today's age-adjusted cancer mortality rates by at least 50% before 2050 (https://www.whitehouse.gov/briefing-room/statements-releases/2022/02/02/fact-sheet-president-biden-reignites-cancer-moonshot-to-end-cancer-as-we-know-it/) (7). National Institute of Health (NIH) supports research on behavioral, biological, and social determinants of health and structural racism and discrimination (https://www.nih.gov/ending-structural-racism/health-equity-research) (8). National Cancer Institute (NCI) starts to unpack Biden's Moonshot 2.0 goal providing new funding opportunities for cancer research in many areas including studies on cancer disparities. The National Cancer Institute has recognized the need of implementing multilevel cancer intervention research to address health disparities and improve population health (9). Several methodological and data analytic challenges of multilevel interventions have been addressed (10). Researchers, institutes, and governments have recognized the importance of integrating social behavioral and biological sciences to effectively and efficiently prevent, detect, and treat cancers (2, 11). To facilitate the adoption of multilevel interventions, it is important for researcher to gain sufficient knowledge on building a multilevel level conceptual framework based on evidence-based theories and models (e.g., theory of planned behavior, socio-ecological model) and develop appropriate study methods (quantitative, qualitative, and mixed methods) and analytical models (e.g., hierarchical modeling) to measure the impact of multilevel interventions on study outcomes (12, 13).

Despite advancements in cancer screening, diagnosis, and treatment, cancer inequalities remain a persistent public health concern in the United States and globally. The papers presented in this Research Topic contribute to the overall literature on cancer inequalities by describing relationships between SDOH and cancer-related outcomes. Further, the studies presented included novel findings regarding the influence of SDOH at multiple levels, which has implications for future research and intervention development. However, additional research is needed to understand the influence of SDOH on diverse communities experiencing cancer inequalities and to identify the pathways by which SDOH impact cancer outcomes to guide the development of strategies to eliminate or reduce cancer-related disparities.

## Author Contributions

T-ST and C-CL contributed to the study conception and drafted the first manuscript. AM reviewed the manuscript and did the interpretation of the discussion. All authors contributed to editing and revising the manuscript critically and approved the final version of the article to be published.

## Conflict of Interest

The authors declare that the research was conducted in the absence of any commercial or financial relationships that could be construed as a potential conflict of interest.

## Publisher's Note

All claims expressed in this article are solely those of the authors and do not necessarily represent those of their affiliated organizations, or those of the publisher, the editors and the reviewers. Any product that may be evaluated in this article, or claim that may be made by its manufacturer, is not guaranteed or endorsed by the publisher.
